# Assemblage of a Semi-Arid Annual Plant Community: Abiotic and Biotic Filters Act Hierarchically

**DOI:** 10.1371/journal.pone.0041270

**Published:** 2012-07-27

**Authors:** Arantzazu L. Luzuriaga, Ana M. Sánchez, Fernando T. Maestre, Adrián Escudero

**Affiliations:** Department of Biology and Geology, Rey Juan Carlos University, Madrid, Spain; University of Alberta, Canada

## Abstract

The study of species coexistence and community assembly has been a hot topic in ecology for decades. Disentangling the hierarchical role of abiotic and biotic filters is crucial to understand community assembly processes. The most critical environmental factor in semi-arid environments is known to be water availability, and perennials are usually described as nurses that create milder local conditions and expand the niche range of several species. We aimed to broaden this view by jointly evaluating how biological soil crusts (BSCs), water availability, perennial species (presence/absence of *Stipa tenacissima*) and plant-plant interactions shape a semi-arid annual plant community. The presence and cover of annual species was monitored during three years of contrasting climate. Water stress acted as the primary filter determining the species pool available for plant community assembly. *Stipa* and BSCs acted as secondary filters by modulating the effects of water availability. At extremely harsh environmental conditions, *Stipa* exerted a negative effect on the annual plant community, while at more benign conditions it increased annual community richness. Biological soil crusts exerted a contradictory effect depending on climate and on the presence of *Stipa*, favoring annuals in the most adverse conditions but showing repulsion at higher water availability conditions. Finally, interactions among co-occurring annuals shaped species richness and diversity of the final annual plant assembly. This study sheds light on the processes determining the assembly of annual communities and highlights the importance of Biological Soil Crusts and of interactions among annual plants on the final outcome of the species assembly.

## Introduction

Disentangling the mechanisms promoting plant coexistence has been a hot topic in ecology for the last decades [1]–[3]. Although there is wide consensus of the primary importance of abiotic filters in community assembly [4]–[6], we are just beginning to understand how biotic interactions (i.e. grazing or plant-plant interactions) restrict or enhance diversity at small scales [7], and how their effects scale up to the community level [8]. Ecological assembly rules (*sensu* [9]) describe restrictions on plant assembly promoted by any of the following ecological filters: dispersal, abiotic environment and biotic interactions. They act in a hierarchical sequence to determine the identity and local abundance of the species co-occurring in a given locality [2], [10]–[12]. Nevertheless, and to our knowledge, no previous study has evaluated under field conditions the hierarchy of abiotic and biotic filters affecting diversity of plant communities.

**Figure 1:**
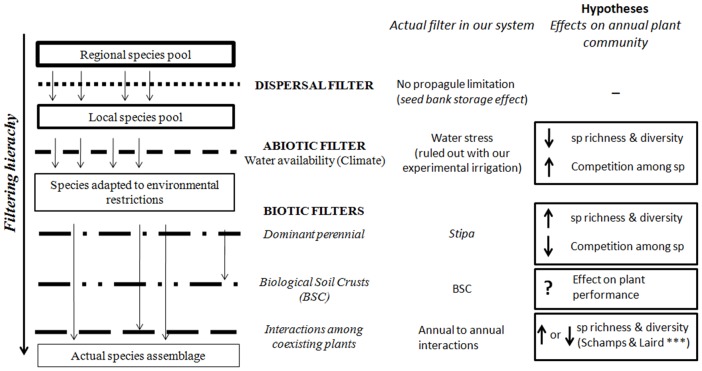
Theoretical framework of our study. It shows the environmental variables acting as abiotic and biotic filters in the studied annual community, and the hypotheses evaluated in this study. The main theoretical framework of our study considers three ecological filters that act in a hierarchical fashion during the species assembly process, the first filter consists on dispersal restrictions that downsizes the species pool from the regional to the local scale [75], subsequently, the available species pool is filtered by the abiotic environment, especially in stressful environmental conditions [60], and finally, the biotic filter that encompasses the intra- and inter-specific interactions mostly act at the plant-to plant spatial scales [76], [77] and define the final species assembly.

Soil water availability, and especially water pulses, largely determine the structure of plant assemblies in arid and semi-arid environments [13], [14], particularly those dominated by annual plants [15], [16]. However, and according to a hierarchical assembly process (Fig. 1), the effects of this primary abiotic filter on semi-arid annual communities are modulated at small spatial scales by biotic filters such as perennial neighbors [17], [18] and biological soil crusts (BSCs; complex combination of bryophytes, lichens and cyanobacteria that are widespread in drylands worldwide; [19]. These crusts strongly affect ecosystem functions such as hydrology and nutrient cycling [20], [21], as well as the establishment and performance of vascular plants [22].

Perennial plants are an important biotic filter shaping the distribution of annual communities. The association of annual species to perennials is common in arid and semi-arid habitats [23]–[25]. Perennials usually ameliorate harsh microclimatic conditions under their canopies [26], [27], and improve soil fertility [28], [29]. Consequently, perennial canopies are usually safer sites for the establishment of annuals [30], leading to denser and more productive fringes of annuals around them compared to adjacent bare ground areas [24]. However, shifts from positive or neutral to negative effects of desert shrubs on annual species with increasing water availability have been described [25], and under extremely dry years, perennials may even out-compete annuals under its canopy [18]. Therefore, climatic conditions are critical on the final outcome of this top-down relationship between perennial nurses and understory annuals.

Together with the effect of perennials and the BSC on annual communities, plant-plant interactions may exert a crucial effect during the assembly process of annual communities [31], [32]. The prevalence of intransitive competition networks among plants are expected to result in more diverse communities; otherwise, if transitive competition networks prevail less diverse communities are expected (see [33], [34]). Plant-plant interactions among annuals are likely to be greatly affected by biotic filters such as perennials. Following the Stress Gradient Hypothesis [35], facilitation among annuals should prevail at open areas where environmental conditions may be very stressful, and annual-annual interactions would turn into competition at the milder environmental conditions existing under the canopy of perennial vegetation; however, other authors consider that when resource limitation is high, neighbors may probably compete for the scarce resources [36].

Thus, if we want to understand the processes that determine the assembly of plant communities, it is essential to evaluate the effect of the primary environmental filter (i.e. climate) together with other biotic filters acting at small spatial scales. Biotic and abiotic filters are likely to interact among them, since perennial plants can strongly affect the composition of BSCs (e.g. [37]), and the later can also affect the physiological performance and nutrient status of plants [38]. Furthermore, both perennial plants and BSCs can also affect plant-plant interactions among annuals.

We evaluated the hierarchy of abiotic (climate) and biotic (perennial plants, BSCs and plant-plant interactions) filters affecting richness (species number/plot) and diversity (inverse Simpson) of annual communities in a Mediterranean semiarid gypsum steppe dominated by *Stipa tenacissima*. Our main objectives were to: 1) address to what extent the climatic abiotic filter (during three consecutive years) determined community assembly processes 2) evaluate how *Stipa* and BSCs affected the annual plant community assembly in the presence and absence of the abiotic filter (by experimentally vanishing its effect thorough irrigation treatments) 3) Explore the role of fine scale annual to annual interactions on final community configuration.

The studied system is especially suitable to explore community assembly processes coexistence mechanisms for different reasons. First, the presence of a dense and quite evenly distributed soil seed bank guarantees the local presence of almost every annual species at any place [39], [40] this means that the dispersal filter is not acting in this system, and consequently, we can attribute the differences in local species assembly to abiotic or biotic filters. Second, the short life-cycle of annuals allows us to cover the whole ontogenetic plant development, avoiding misleading results related to changes in the magnitude and direction of interactions along different life-stages of the co-occurring plants [41], [42]. Even more, it is well known that the presence of *Stipa* usually affects BSC composition and structure [37], and there are also evidences that BSCs affect water use efficiency of *Stipa* tussocks [38]. Finally, in this study we have ruled out the effect of the abiotic filter by experimentally increasing water availability.

## Methods

All necessary permits were obtained for the described field study. The environmental ministry of the Madrid Autonomous Region issued the permission for the studied location in Aranjuez. Field studies did not involve endangered or protected species.

### Study Site

The study was conducted at the Aranjuez experimental station, which is located in central Spain (40°02′N–3°37′W; 590 m.a.s.l.). Climate is semi-arid Mediterranean with mean annual rainfall of 400 mm/m^2^ year^−1^, and mean annual temperature of 14.5°C. The soil is derived from gypsum outcrops, and is classified as Xeric Haplogypsid [43]. Aboveground vegetation is a semi-arid gypsum steppe mainly dominated by *Stipa tenacissima*, a tussock-forming grass, scattered in a bare ground and BSC matrix dominated by crustose lichens (e.g. *Diploschistes diacapsis*, *Squamarina lentigera*, *Fulgensia subbracteata*, *Toninia sedifolia* and *Psora decipiens*), and by a very rich annual plant community of up to 38 species/0.25 m^2^ (e.g. *Chaenorrhinum reyesii, Ctenopsis gypsicola*, *Campanula fastigiata* and *Sedum gypsicola*).

### Experimental Design and Monitoring

Surveys were completed along the spring seasons of 2008, 2009 and 2010. A weather station was located at the study area in order to monitor temperature and rainfall during this period (Figure S1).

Twenty four 50×50 cm plots were randomly located in the two most common microenvironments present at our study site: 12 plots were located below mature *Stipa* plants and 12 in open areas devoid of perennial vegetation (located at least 1 m apart from the nearest *Stipa* tussock). Each plot was divided into 100 cells of 5×5 cm, which were our sampling units (2,400 sampled cells). This grid cell size was chosen to be in concordance with the average size of the annual species studied, which are usually under 5 cm diameter, to be sure that co-occurring species are really interacting. From the 2008th to the 2010th spring seasons, every annual plant species cover was registered at each cell. The cover of every lichen species was also estimated at each cell in 2008.

In 2008 and 2009, half of the plots were irrigated with an amount equivalent to 28 l/m^2^ evenly distributed in seven irrigation events along February and March, when most annual plants develop from the seedling stage and reach reproductive maturity. To minimize potential edge effects, a buffer of 20 cm around each plot was irrigated as well. Our irrigation treatment supposed a meaningful increase (more than the 50%) comparing with the natural rainfall recorded in the last 30 years for the same time lapse. With this treatment we aimed to remove potential constrains to the germination and development of annuals imposed by lack of water (abiotic filter). The 2010 spring was extremely rainy, 2.4 times rainier than the last 30 years for the same time lapse, so we did not conduct our irrigation treatment.

### Diversity Indices

We calculated the inverse Simpson index to estimate the diversity of annuals and of the BSCs at each plot for the three sampling years [44]:

Inverse Simpson  = 1/(∑P_i_^2^).

This index has also been called the “effective number of species” [45], and it represents the number of species that a community would have if the species were equally distributed. Diversity indices of annuals and lichens at the plot level were independently calculated with PRIMER v.6.1.11 software.

### Spatial Patterns of Annuals and Biological Soil Crusts

In order to evaluate the spatial distribution of the annual and BSC communities in each plot we used the Spatial Analysis by Distance Indices approach (SADIE) [46], [47], which has successfully been used to characterize the spatial pattern of BSCs and vegetation in *Stipa* steppes [48], [49]. This approach allows hypothesis testing for the presence of a spatial pattern in the form of clustering into patches and into gaps of a given variable. The index of aggregation (I_a_) measures the overall aggregation in each plot, the larger the I_a_, the more spatially aggregated is the observed variable [50]. Aggregation indices close to unity mean that the spatial distribution is nearly random. Significance is obtained by means of a randomization procedure that consists in the permutation of the values among all sample units. We calculated I_a_ separately for richness and total cover data for each plant matrix (72 matrices  = 24 plots × 3 years), and richness and cover for lichen matrices (24 matrices). Cover values were transformed into 10 categories in order to fit to the statistical requirements needed to apply the analyses [46], [50].

Two particular data sets may be spatially positively associated (coincidence of patches and gaps of both data sets), negatively associated (opposite cluster types) or occur at random with respect to one another [50]. Local spatial association between annual plants and lichens in terms of species richness and cover of each biotic component was measured using the association index (χ_i_) provided by SADIE [51]. This index is based on the similarity between the clustering indices of both communities (lichens and annuals); positive values of this index arise from coincidence of patches or gaps in both data sets, and negative values from opposite cluster types. Values of χ_i_ vary between −1 (variables spatially dissociated) and +1 (variables spatially associated). Significance of χ_i_ was tested by randomizations and lately corrected with the Dutilleuĺs method [52] in order to account for spatial autocorrelation [51]. Associations are significant when p<0.025 and dissociations are significant when p>0.975. Values of χ_i_ were calculated for each combination of lichen and annual species richness and cover.

### Null Model Approach to Test Community Level Interactions

To estimate the outcome of biotic interactions among annuals at the community level, we carried out null model analyses of co-occurrence patterns [53]. Null-model analyses consist in comparing observed co-occurrences between sampled communities and simulated stochastic communities. This approach has been used to evaluate the importance of competitive and facilitative interactions in shaping plant and lichen assemblies [54]–[56]. We acknowledge that species co-occurrence can be affected by processes other than biotic interactions, such as habitat selection and limited dispersal [1]. However, we believe that these aspects can only marginally affect co-occurrence in the studied communities because the characteristics of our sampling design (which minimized the amount of non-suitable habitat sampled), and the dispersal characteristics of the species studied (which make them quite unlikely to be dispersal-limited). We used the C-score index [57] to quantify the pattern of co-occurrence of plant species. This index has been proven to be less sensitive to the presence of noise in the data, and has good statistical properties [53]. The C-score is calculated for each pair of species as (R_i_-S)(R_j_-S), where R_i_ and R_j_ are the number of total occurrences for species i and j, and S is the number of squares in which both species occur; this score is then averaged over all possible pairs of species in the matrix [57]. The larger the C-score value of a given matrix, the lower the species co-occurrence in that matrix. In a competitively structured community, the C-score is expected to be significantly larger than expected by chance.

A total of 72 presence-absence matrices (24 plots × 3 years), were built. We analyzed them using a fixed-equiprobable null model. This null model treats the row sums (species) as fixed and the columns (sites) as equiprobable. This means that the simulated communities were randomly created by re-shuffling species occurrences among all samples, always maintaining the species number fixed, being the total presence of each species the same in all the null models generated. This restriction keeps species specific abundance and rarity in the null models similar to the original. Maintaining sites as equiprobable in this null model assumes, as well, that all the sites are equally suitable for all species, and its use has been recommended when dealing with standardized surveys conducted in homogeneous habitats [53]. We used 50,000 swaps to generate the null distribution in order to overcome high type I error rates reported for large matrices [58]. In 2008, two non-irrigated plots in the *Stipa* treatment showed less than two species, so null models could not be run on neither of these two plots. We used Ecosim 7.72 software to perform these analyses.

In order to compare the C-score indices of the 72 plots, we calculated the Standardized Effect Size (SES) of each matrix by standardizing the difference between the observed and the simulated models: SES  = (I_obs_ – I_sim_)/σ_sim_. The SES measures in terms of standard deviation units the departure of an observed index beyond or below the average index of the simulated matrices [59]. To test if these indices were significantly different from zero, studentś t-tests were performed for each irrigation treatment and microenvironment.

### Statistical Analyses

#### Repeated measures generalized linear models

We performed repeated measures Generalized Linear Models (rm-GLM) to model six attributes of the annual plant community: richness, Inverse Simpson index, total cover, I_a_ carried out with richness data, I_a_ carried out with cover data and the SES of the C-score (hereafter Cscore-SES). Note that for all these variables we have a unique value for each plot and year. We built a fully factorial model for each variable, with year as the repeated measures factor, and microenvironment and irrigation as fixed factors. To evaluate the effect of BSCs on annual community properties we used two variables summarizing the diversity and spatial structure of the lichen community: the lichen Inverse Simpson index and the lichen cover aggregation index. We checked all these variables to avoid multicollinearity problems. The error distribution and link function that better fitted our data were used for each rm-GLM (Table 1).

**Table 1:** Summary of repeated-measures Generalized Linear Models conducted with six features of the annual plant communities studied.

	*Richness*	*Inverse Simpson*	*Cover*	*I_a_ richness*	*I_a_ cover*	*Cscore-SES*
Distr.	N	N	N	N	N	N
Link	Id.	Log.	Id.	Log	Log	Id.
**Intercept**	1.9	3.6	6.8	8.5	6.4	1.8
**Year**	**654.3** ***	**19.8** ***	**16.6** ***	**15.4** ***	**1.03**	**115.3** ***
**Microenv**	0.1	0.9	**5.5** *	0.5	1.2	0.05
**Irrigation**	**4.5** *	**8.3** **	0.15	1.5	1.7	**6.0** *
**Year** * **Micr**	**20.3** ***	**16.7** ***	1.6	2.5	1.3	**9.6** **
**Year** * **Irri**	2.2	3.5	3.6	**9.5** **	0.6	3.1
**Micr** * **Irri**	2.1	3.4	1.5	0.1	0.01	**6.2** *
**Year** * **Micr** * **Irri**	5.1	0.1	2.7	4.1	0.4	0.5
*Lichen community*						
**Cover aggr. index**	0.2	0.1	**5.4** *	**12.6** ***	3.0	0.02
* Coef ±S.E.*	*−0.7±1.5.*	*0.04±0.12*	***−1.6±0.7***	***0.15±0.04***	*0.1±0.06*	*0.03±0.2*
**Inverse Simpson**	0.3	3.1	**5.1** *	2.1	2.7	3.3
* Coef ±S.E.*	*0.4±0.7*	*0.09±0.05*	***0.8±0.3***	*−0.03±0.02*	*−0.05±0.03*	*3.4±2.2*

Error distributions (Distr) and link functions (Link) assumed in the GLM are shown. Walds Chi-square and significance levels are indicated. *Cscore-SES*: Standardized Effect Size of the C-Score values, N: Normal, Id.: identity link function, Log: Logarithmic link function, *I_a_*  =  index of aggregation, Coef: coefficient, and S.E.: Standard Error.

* 0.01<*p*<0.05;

** 0.001<*p*<0.01;

*** *p*<0.001.

The values of χ_i_ evaluating the degree of association between the spatial patterns of richness and cover of both BSCs and annuals (four pair wise association indices) were also modeled by means of rm-GLM by considering microenvironment and irrigation as fixed factors.

## Results

The autumn-spring period previous to the 2008 sampling was extremely dry; the total autumn precipitation was 79 mm/m^2^, from which 56 mm/m^2^ fell in a single day. Winter and early spring were even drier, with only 38.5 mm/m^2^ (hereafter called “the dry year”). On the contrary, the autumn previous to the 2009 sampling was significantly wetter (160.6 mm/m^2^) and followed by a dry winter (77.5 mm/m^2^; hereafter called “wet-dry year”). In 2010, the opposite rainfall distribution happened, with a relatively dry autumn followed by a very wet winter (40.3 mm/m^2^ and 343.6 mm/m^2^, respectively, hereafter called “dry-wet year”).

### Diversity and Spatial Pattern of the Annual Community

**Figure 2:**
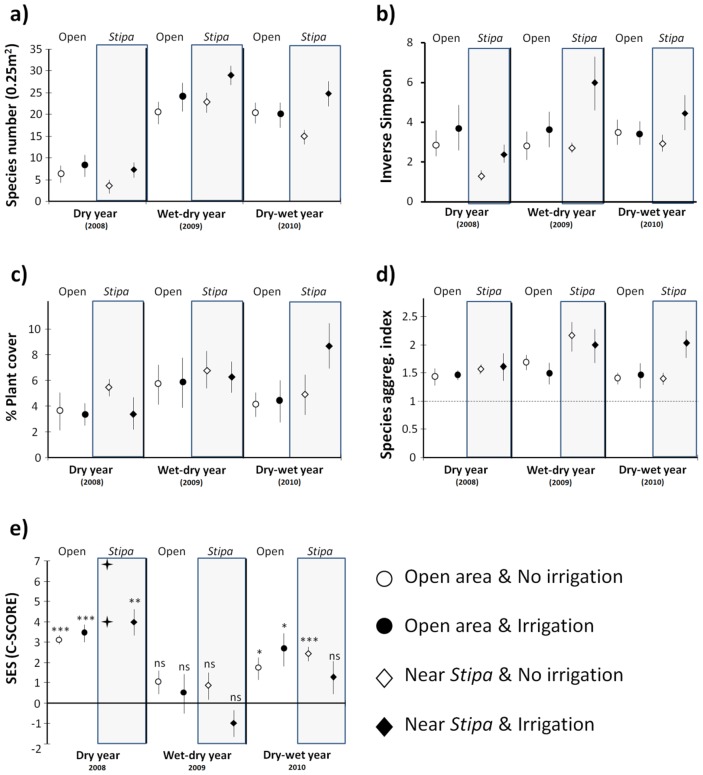
Effect of the experimental treatments on the annual plant community attributes. Mean values of species richness (a) the inverse Simpson diversity index (b), plant cover (c), species aggregation index (d), and the SES of the C score (e) of the annual community along the three study years and in the four experimental scenarios. Vertical bars represent standard errors. Horizontal dashed lines in **d** represent the threshold below which aggregation is not different from random; in **e** T-test was performed with the Cscore-SES values of each treatment (n = 6) in order to test significant differences from cero. +: SES values of two individual plots (see methods). ***: p<0.001; **: p<0.01; *: p<0.05; ns: not significant.

We found 71 annual species in our plots during the three years of our study (Table S1). Inter-annual variability was the greatest source of variation in this ephemeral community (Table 1; Fig. 2). Plant species richness varied from 33 species in the driest year (2008) to 64 species in the wet-dry year (2009). Irrigation significantly increased richness and diversity, but had no significant effects on total plant cover or spatial pattern. *Stipa* tussocks increased the cover of annuals, and a microenvironment × year interaction was observed when evaluating species richness and diversity data; in the dry year, richness and diversity were significantly reduced adjacent to *Stipa* tussocks, while in the wet-dry year the opposite pattern was found (Figs. 2a and 2b). Plant species were aggregated in our study system in the three study years (Fig. 2d), but the degree of aggregation varied depending on the year and irrigation treatment; in the wet-dry year, irrigated plots showed less aggregation than control plots, while in the dry-wet year (2010), the highest aggregation occurred adjacent to irrigated *Stipa* plants.

### Plant-plant Interactions Among Annuals

Overall, species interactions among annuals were mainly competitive, and they were primarily determined by the year and irrigation treatments and, to a lesser extent, by their interaction with microenvironment (Table 1; Fig. 2e). The most intense competitive interactions among co-occurring annuals were found in the driest year, with irrigation and the presence of *Stipa* slightly increasing species interference. In the wet-dry year, the SES values were not significant but in the dry-wet year, species interference was reduced in the irrigated plots adjacent to *Stipa*, comparing with irrigated open areas. A significant negative correlation between the C score-SES and species richness was also observed (Rho = *−*0.75; p<0.001; n = 65).

### Biological Soil Crust Effects on the Annual Community Assembly

**Figure 3:**
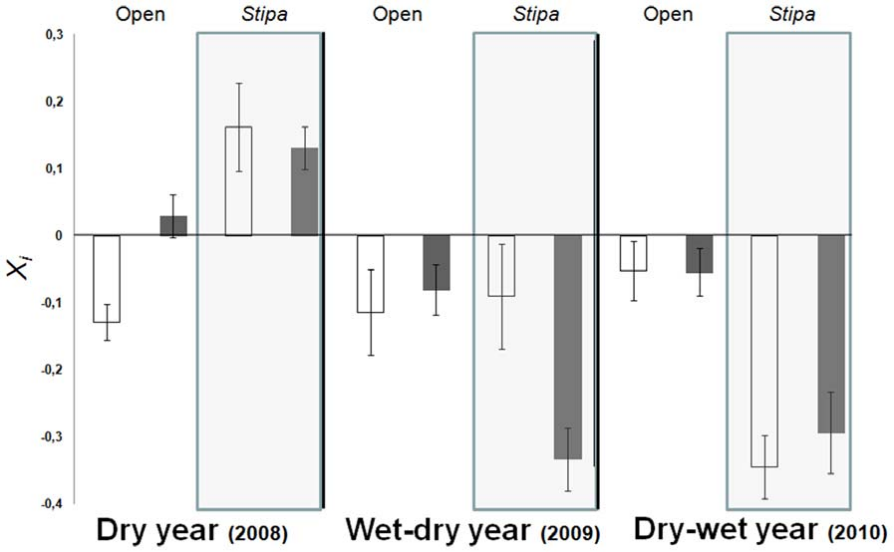
Effect of the experimental treatments on the relationship between the annual plant and the BSC. Mean values of the association index (*χ_i_*) between lichen cover and plant cover along the three study years and in the four experimental scenarios. Data represent means ± standard errors. n = 72.

The diversity and the I_a_ of BSCs affected the total cover and the I_a_ of species richness of the annual community (Table 1). No significant effects of BSCs on plant richness, diversity or on plant-plant interactions were detected (Table 1). Increases in the aggregation of BSCs led to reductions and increases in the cover and I_a_ of richness of annuals, respectively. In addition, BSC diversity exerted a positive effect on plant cover. Association indices between lichen and plant communities (Table 2; Fig. 3) showed that, in the driest year, a spatial association between the cover of BSCs and annuals occurred adjacent to *Stipa*, whereas that in the wetter years spatial dissociation between lichen and plant communities prevailed, especially under *Stipa* canopies.

**Table 2:** Generalized Linear Models with the association indices (X-index) of lichen and plant richness and covers in the three sampling years.

	Association index (X-index)			
	Lichen richness		Lichen cover	
	Plant richness	Plant cover	Plant richness	Plant cover
**Intercept**	1.7	12.5	2.6	10.9
**Year**	4.2	**30.3** ***	**21.2** ***	**39.4** ***
**Microenvironment**	0.001	1.0	0.001	1.1
**Irrigation**	0.01	0.02	1.4	0.01
**Year** * **Microenv**	**10.6** ***	**38.8** ***	**28.9** ***	**31.3** ***
**Year** * **Irrigation**	1.9	**8.1** *	**12.2** **	5.5
**Microenv** * **Irrigation**	0.2	0.9	3.6	1.3
**Year** * **Microenv** * **Irrig**	0.3	**7.4** *	0.3	3.8

GLMs were performed assuming normal distributions of errors and with the identity link function. Walds Chi-square and significance levels are indicated.

* 0.01<*p*<0.05;

** 0.001<*p*<0.01;

*** *p*<0.001.

## Discussion

Our results support the theoretical framework proposed in this study to explain assembly process of annual plant communities in semi-arid systems (Fig. 1). As expected, climate was the primary abiotic filter shaping annual plant communities [15], [16], and under the harsh environmental restrictions imposed by drought, the assembly of annuals was shaped to a great extent by *Stipa* and by BSCs. In this sense, we agree with other recent studies that found that when stressful abiotic filters restrict the species pool ready to conform local communities, niche assembled communities prevailed [60], [61].

*Stipa* tussocks ameliorated environmental conditions for annuals, inducing higher plant productivity (in terms of cover). Contrarily to our expectations, *Stipa* did not have positive effects on richness and diversity. These results do not agree with the findings of other studies where nurse plants increased richness by broadening specieś ecologically suitable microhabitats and promoting microhabitat segregation among them [62]. This study suggests that *Stipa* plants largely determined assembly of the annual plant community, even in the absence of the abiotic filter in irrigation treatments. This implies that the microenvironmental conditions created by *Stipa* (e.g. reduction of radiation, enhancement of soil moisture, increased soil fertility) [27], [63] exerted a filtering effect on the local annual plant community independently of climate. Thus, although the number of species in the metacommunity was really high (70 annuals) and in spite of the lack of propagule limitation in our system (because seed bank dynamics guarantee seed availability everywhere, see [40]), differences in local species assembly between both microenvironments are the result of the action of subsequent hierarchical filters.

Although the widely established idea that perennials act as nurses and are the main drivers of facilitative interactions in arid ecosystems, our findings showed a different scenario. The effect of *Stipa* on local annual communities largely shifted depending on water conditions. In the driest year, we did not observe any facilitation effect of *Stipa* on the annual community; indeed a reduction in species richness and diversity was observed close to tussocks, suggesting negative interaction between *Stipa* and annual species. Our study also supports the findings of Holmgren and Scheffer [64], who argued that facilitative interactions with perennials should prevail under intermediate stress conditions, and that at extremely harsh environments nurse plants may be unable to mitigate stressful conditions for their neighbors [7], [65]. Negative effects of *Stipa* on annual plant assemblies have been reported in other studies in semi-arid systems, and it has been related to the belowground depletion of water availability in the zone of influence of *Stipa* [18]. When water stress conditions were alleviated (in the wetter years 2009 and 2010, and in the irrigation treatments), *Stipa* exerted a net facilitative effect on the annual plant community that increased species richness and diversity. Our results suggest that water stress acted as an environmental filter restricting the species pool available to establish. Consequently, when water shortage was alleviated (naturally or experimentally), more species from the species pool were ready to conform the local community assembly. The larger the species pool available, the more species may find their realized niche under the milder microenvironmental conditions close to *Stipa*. This would result in a facilitation effect of *Stipa* plants on the annual community at medium environmental severity levels [64].

Over-imposed on the filtering effect of perennials, our findings suggest that plant-plant interactions among annuals are key drivers of local community assembly in low productivity stressful environments [66]. In the dry year, species assemblies were mainly shaped by intense competitive interactions among annuals (see also [67]), while in more benign conditions species seemed to be randomly assembled. We did not observe facilitation among annual plant species in any of the scenarios analyzed in our study. These results contradict the hypothesis that in stressful environments positive interactions prevail and competition plays a minor role in plant performance [35], [68]. Instead, we provide experimental support for the hypothesis suggesting that shifts from competition to facilitation with increasing stress are less likely if belowground competition for a limited resource is expected, such as in semiarid environments [7], [36], [69]. Intense interference among annuals in semiarid environments may also occur due to high resource limitation [10], [70].

We also found that *Stipa* affected the competitive outcome of their understory annual plants comparing to open areas. This effect was strictly linked to yearly climatic conditions reinforcing again the prevalence of hierarchically acting filters in stressful environments. In the driest year, *Stipa* most likely emphasized resource restriction for annuals leading to higher competitive interactions among them, especially in not irrigated plots. Our results suggest that plant-plant interactions should be also considered during species assembly in a hierarchical filtering context if we want to scale up the role of plant-plant interactions to the community level [8]; thus, interactions with perennials, which are the norm in these systems, are concurrent with interactions at a smaller spatial scale to conform the assembly of annual communities. Facilitative interactions at the first level (perennials to annuals) are not necessarily followed by positive interactions at the lower level (annual to annual interactions).

We detected that the increase in competition intensity among annuals was closely related to a decrease in annual species richness at the plot level (Rho = *−*0.75; p<0.001). This suggests that a small number of annual species was able to out-compete the others and consequently, when competition among annuals was more intense, species richness locally decayed. This implies that the annual plant species in our system were probably hierarchically structured in terms of competition intensity [34], [71].

The effect of BSCs on the annual assembly was also very significant, but of less intensity than that of *Stipa*. Although BSCs did not affect plant species richness or diversity, it significantly determined annual plant cover and species composition (data not shown). Specifically, the most conspicuous lichens (*Diploschistes*, *Fulgensia* and *Squamarina*) were the main responsible for this effect. The mechanisms underlying this outcome are still not well-known. Two possible mechanisms acting isolated or jointly have recently been experimentally demonstrated [22]. First, the lichen community exhibit high chemical complexity, so there could be a large amount of allelopathic compounds not only for lichens but probably also for annuals [72]; secondly, differences in morphology may strongly influence the redistribution of water resources, which could lead to facilitate annual species germination and establishment. For example, an unbroken lichen crust such as *Diploschistes* is likely to shed water like a patch of exposed bedrock [73], whereas the hydrological behavior of species like *Cladonia* or *Squamarina* could generate a very favorable environment for early life stages of annuals [7]. These features suggest beneficial or detrimental effects on annual plants that could be species-specific [22].

Furthermore, the modulating effect of both *Stipa* and BSCs shifted from year to year. In general terms, we found that BSC and annual covers tended to be dissociated close to *Stipa*, but in the driest year this relationship shifted dramatically, and we observed positive associations of BSC and annual plant cover adjacent to *Stipa*. This may indicate that, in dry years, at the most stressful drought conditions, close to *Stipa* plants [74], lichens may favor annual species establishment, probably due to water soil moisture lasting for longer in the soil below BSCs [20]. This effect may shift into dissociation between both components, most likely due to competition for living space during more beneficial environmental conditions in wetter years.

### Conclusions

Our findings indicate that our theoretical framework, in which a set of abiotic and biotic filters act in a hierarchical way, can shed light on the assembly process of annual communities in semiarid environments and on plant coexistence patterns in general. Thus, climate constitutes the main filter determining the assembly of annual communities; water availability acted as the primary environmental filter and most likely determined the available species pool. This primary filter is modulated by the interaction with local biotic filters, such as the effect of perennials and BSCs and their interactions which shifted depending on water availability. At harsh conditions, *Stipa* exerted a negative effect on the annual plant community, but as environment became milder the effect of *Stipa* increased the realized niche of several species, resulting in richer and more diverse annual communities. The lichen community exerted a contradictory effect depending on climate (i.e. water availability) and the presence of *Stipa*, favoring the presence of annuals in the most adverse conditions but showing repulsion patterns at higher water availability conditions. Finally, interactions among co-occurring annuals shaped species richness and diversity of the final annual plant assembly. This study sheds light on BSCs as important determinants of semi-arid annual communities, and on the processes determining the assembly of these communities.

## Supporting Information

Figure S1 **Daily precipitation and maximum temperatures during autumn and winter in the three sampling years.** Data registered in a meteorological station placed in the study area (Aranjuez, Madrid, Spain). **a)** 2008 sampling year was very dry, the first rain event happened late (October the 3^rd^ 2007) and it was torrential (nearly 60 mm/m^2^). **b)** 2009 showed a very rainy autumn and a dry winter. **c)** 2010 was characterized by a very dry autumn followed by an extremely rainy winter. Horizontal dotted lines represent the irrigation period (seven irrigation events/year). Black arrows indicate the beginning of the annual plant community sampling.
(TIF)

Table S1 **List of the plant and lichen species occurring in our study area.** Abbr.: abbreviation, LC: Life cycle: Gypso: gypsophily; A: annual; P: perennial. S: strict gypsophyte O: optional gypsophyte.
(DOCX)
