# Supplementary material for: Assemblage of a Semi-Arid Annual Plant Community: Abiotic and Biotic Filters Act Hierarchically
Source: PLoS One. 2012 Jul 27;7(7):e41270. doi: 10.1371/journal.pone.0041270 (PMC3407229; doi:10.1371/journal.pone.0041270)
Supplement: Table S1 — List of the plant and lichen species occurring in our study area. Abbr.: abbreviation, LC: Life cycle: Gypso: gypsophily; A: annual; P: perennial. S: strict gypsophyte O: optional gypsophyte. (DOCX) [file pone.0041270.s002.docx]

**Table S1**.

| **Abbr** | **Species** | **Family** | **LC** | **Gypso** |
| --- | --- | --- | --- | --- |
| allisph | *Allium sphaerocephalon* L. | Liliaceae | P | O |
| alysim | *Alyssum simplex* Rudolphi | Cruciferae | A | O |
| anaarv | *Anagallis arvensis* L. | Primulaceae | A | O |
| arelep | *Arenaria leptoclados* (Rchb.) Guss. | Caryophyllaceae | A | O |
| aspram | *Asphodelus ramosus* L. | Liliaceae | P | O |
| astaqu | *Asteriscus aquaticus* (L.) Less | Compositae | A | O |
| astlin | *Asterolinon linum-stellatum* (L.) Duby in DC. | Primulaceae | A | O |
| bromus | *Bromus rubens* L. | Gramineae | A | O |
| cameri | *Campanula erinus* L. | Campanulaceae | A | O |
| camfas | *Campanula fastigiata* Dufour ex A. DC. | Campanulaceae | A | S |
| cenhys | *Centaurea hyssopifolia* Vahl | Compositae | P | S |
| cenmel | *Centaurea melitensis* L. | Compositae | A | O |
| cencal | *Centranthus calcitrapae* (L.) Dufr. | Valerianaceae | A | O |
| cerglo | *Cerastium glomeratum* Thuill. | Caryophyllaceae | A | O |
| cerpum | *Cerastium pumilum* Curtis | Caryophyllaceae | A | O |
| charey | *Chaenorrhinum reyéis* (C. Vicioso & Pau) Benedí | Scrophulariaceae | A | S |
| clyjon | *Clypeola jontlaspi* L. | Cruciferae | A | O |
| Cresp | *Crepis sp* | Compositae | A | O |
| ctegyp | *Ctenopsis gypsophila* (Hack). Paunero | Gramineae | A | S |
| dipser | *Dipcadi serotinum* (L.) Medik | Liliaceae | P | O |
| dipvim | *Diplotaxis viminea* (L.) DC. | Cruciferae | A | O |
| echcre | *Echium creticum* L. | Boraginaceae | A | O |
| erocic | *Erodium cicutarium* (L.) L´Her. | Geraniaceae | A | O |
| eropul | *Erodium laciniatum* subsp. p*ulverulentum* (Boiss.) Batt. | Geraniaceae | A | O |
| erover | *Erophila verna* (L.) Chevall | Cruciferae | A | O |
| eupexi | *Euphorbia exigua* L. | Euphorbiaceae | A | O |
| eupfal | *Euphorbia falcata* L. | Euphorbiaceae | A | O |
| eupsul | *Euphorbia sulcata* De Lens ex Loisel | Euphorbiaceae | A | O |
| filpyr | *Filago pyramidata* L. | Compositae | A | O |
| galpar | *Galium parisiense* L. | Rubiceae | A | O |
| gerpus | *Geranium pusillum* L. | Geraniaceae | A | O |
| gypstr | *Gypsophila strutium* (L). in Loefl | Caryophyllaceae | P | S |
| helsal | *Helianthemum salicifolium* (L.) Miller. | Cistaceae | A | O |
| helsqu | *Helianthemum squamatum* (L.) Dum. Cours. | Cistaceae | P | S |
| hercin | *Herniaria cinerea* DC. in Lam. & DC. Fl | Caryophyllaceae | A | O |
| hippcil | *Hippocrepis ciliata* Willd. in Ges. Naturf. | Fabaceae | A | O |
| holoumb | *Holosteum umbellatum* L. | Caryophyllaceae | A | O |
| horpet | *Hornungia petraea* (L.) Rchb. | Cruciferae | A | O |
| hypocho | *Hypochoeris* | Compositae | A | O |
| laupum | *Launaea pumila* (Cav.) Kuntze | Compositae | P | O |
| Leon | *Leontodon* | Compositae | A | O |
| lepsub | *Lepidum subulatum* L. | Cruciferae | P | S |
| limech | *Limonium echioides* (L.) Mill | Plumbaginaceae | A | O |
| lingla | *Linaria glauca* (L.) Chaz | Scrophulariaceae | A | O |
| malaeg | *Malva aegyptia* L. | Malvaceae | A | O |
| medmin | *Medicago minima* L. | Fabaceae | A | O |
| mibmin | *Mibora minima* (L.) Desv | Gramineae | A | O |
| minham | *Minuartia hamata* (Hausskn. & Bornm.) Mattf. | Caryophyllaceae | A | O |
| minmed | *Minuartia editerránea* (Ledeb.ExLink) K. Malý | Caryophyllaceae | A | O |
| neaapu | *Neatostema apulum* (L.) I. M. Johnston | Boraginaceae | A | O |
| parlat | *Parentucellia latifolia* (L.) Caruel | Scrophulariaceae | A | O |
| pishis | *Pistorinia hispanica* (L.) DC. | Crassulaceae | A | O |
| plaafra | *Plantago afra* L. | Plantaginaceae | A | O |
| plaspi | *Platycapnos spicata* (L.) Bernh. | Papaveraceae | A | O |
| poabul | *Poa bulbosa* L. | Gramineae | P | O |
| ranpal | *Ranunculus paludosus* Poir. | Ranunculaceae | P | O |
| resphy | *Reseda phyteuma* L. | Resedaceae | A | O |
| resstr | *Reseda stricta* Pers. | Resedaceae | A | S |
| ressuf | *Reseda suffruticosa* Loefl. Ex Koelp. In Loefl. | Resedaceae | P | S |
| retsph | *Retama sphaerocarpa* (L.) Boiss | Leguminosae | P | O |
| sedgyp | *Sedum gypsicola* Boiss. & Reuter | Crassulaceae | P | S |
| senaur | *Senecio gallicus* Chaix. | Compositae | A | O |
| sherarv | *Sherardia arvensis* L. | Rubiaceae | A | O |
| stiten | *Stipa tenacissima* L. | Gramineae | P | O |
| stodic | *Stoibrax dichotomum* Raf. | Umbeliferae | A | O |
| sisrun | *Sisymbrium runcinatum* Lag. Ex DC. | Cruciferae | A | O |
| tolnod | *Tolpis nodosa* (L.) Gaertn. | Cruciferae | A | O |
| triloe | *Trisetum loeflingianum* (L.) C.Presl | Gramineae | A | O |
| valcor | *Valerianella coronata* (L.) DC. | Valerianaceae | A | O |
| vulcil | *Vulpia ciliata* Dumort. | Gramineae | A | O |
| Zizhis | *Ziziphora hispanica* L. | Labiatae | A | O |
| **Lichen species** | |  |  |  |
| Acanod | *Acarospora nodulosa* (Dufour) Hue | Acarosporaceae |  | |
| Buelli | *Buellia* sp. | Physciaceae |  | |
| Clacon | *Cladonia convoluta* (Lam.) Anders | Cladoniaceae |  | |
| Colcri | *Collema crispum* (Huds.) Weber ex Wigg. | Collemataceae |  | |
| Dipdia | *Diploschistes diacapsis* (Ach.) Lumbsch | Thelotremataceae |  | |
| Fulsub | *Fulgensia subbracteata* (Nyl.) Poelt | Teloschistaceae |  | |
| Psodec | *Psora decipiens* (Hedw.) Hoffm | Psoraceae |  | |
| Squasp | *Squamarina* spp. | Stereocaulaceae |  | |
| Tonsed | *Toninia sedifolia* (Scop.) Timdal | Bacidiaceae |  | |
